# Identification of Autophagy-Related Genes in the Potato Psyllid, *Bactericera cockerelli* and Their Expression Profile in Response to ‘*Candidatus* Liberibacter Solanacearum’ in the Gut

**DOI:** 10.3390/insects12121073

**Published:** 2021-11-30

**Authors:** Xiao-Tian Tang, Cecilia Tamborindeguy

**Affiliations:** Department of Entomology, Texas A&M University, College Station, TX 77843, USA; xiaotian.tang@yale.edu

**Keywords:** autophagy, *Candidatus* Liberibacter solanacearum, *Bactericera cockerelli*, gut, gene expression

## Abstract

**Simple Summary:**

In North America, the bacterial plant pathogen ‘*Candidatus* Liberibacter solanacearum’ (Lso) infects solanaceous plants. Currently, Lso haplotypes, LsoA and LsoB are transmitted by potato psyllid, *Bactericera cockerelli* (Šulc). Because these bacteria are transmitted in a circulative and persistent manner, the gut of the psyllid is the first organ they encounter and could be a barrier to its transmission. Therefore, it is important to understand the molecular mechanisms involved in Lso acquisition and transmission. This study explored if an autophagic response was triggered in response to LsoA and/or LsoB in the gut of the adult potato psyllid. The results showed that Lso may induce the autophagic response in the adult psyllid gut since the majority of autophagy-related genes (ATGs) are sensitive and responsive to the exposure or infection of both LsoA and LsoB. Therefore, this study represents a stepping-stone towards understanding the molecular mechanisms involved in Lso transmission.

**Abstract:**

Autophagy, also known as type II programmed cell death, is a cellular mechanism of “self-eating”. Autophagy plays an important role against pathogen infection in numerous organisms. Recently, it has been demonstrated that autophagy can be activated and even manipulated by plant viruses to facilitate their transmission within insect vectors. However, little is known about the role of autophagy in the interactions of insect vectors with plant bacterial pathogens. ‘*Candidatus* Liberibacter solanacearum’ (Lso) is a phloem-limited Gram-negative bacterium that infects crops worldwide. Two Lso haplotypes, LsoA and LsoB, are transmitted by the potato psyllid, *Bactericera cockerelli* and cause damaging diseases in solanaceous plants (e.g., zebra chip in potatoes). Both LsoA and LsoB are transmitted by the potato psyllid in a persistent circulative manner: they colonize and replicate within psyllid tissues. Following acquisition, the gut is the first organ Lso encounters and could be a barrier for transmission. In this study, we annotated autophagy-related genes (ATGs) from the potato psyllid transcriptome and evaluated their expression in response to Lso infection at the gut interface. In total, 19 ATGs belonging to 17 different families were identified. The comprehensive expression profile analysis revealed that the majority of the ATGs were regulated in the psyllid gut following the exposure or infection to each Lso haplotype, LsoA and LsoB, suggesting a potential role of autophagy in response to Lso at the psyllid gut interface.

## 1. Introduction

Autophagy, which means “self-eating”, is a conserved cellular degradation process that serves to deliver cytoplasmic proteins and organelles to lysosomes for degradation [1]. It plays important roles in many biological processes, such as maintaining homeostasis and preventing nutritional and metabolic-mediated stresses in eukaryotic organisms ranging from yeast to insects and mammals [2]. Autophagy is also an important component in the innate immune defense against infection by viral, bacterial, and fungal pathogens in animals [3]. 

In the last two decades, a series of autophagy-related genes (ATGs) have been characterized from lower (e.g., yeast) to higher eukaryotes (e.g., mammals and insects) [4,5,6]. Most but not all the ATGs play roles in the canonical autophagy pathway, regulating the formation of the autophagosome, a vesicular structure that is the hallmark of autophagy [7]. In yeast, more than 30 ATGs were identified, and around 20 of those are required for the efficient formation of sealed autophagosomes that proceed to fuse with lysosomes. In *Drosophila*, nearly all the core autophagy genes function in the autophagy pathway [6,8,9]. Under normal conditions, autophagy is kept at a low level, but it can be activated by starvation or other stress signals, which are known to induce the expression of some ATG genes [10]. Therefore, the identification of genes that participate in autophagy should establish a foundation for furthering our understanding of the molecular mechanism and the potential roles of autophagy in different physiological processes.

In insects, previous studies have revealed that autophagy components are important for *Drosophila* defense against viruses, such as vesicular stomatitis virus [11]. An increasing amount of evidence shows that autophagy could be induced also by plant pathogens in hemipteran insects, in particular by vector-borne plant viruses. The induced autophagy may play different roles: it could be a defensive mechanism to protect the insect against the invasion of the pathogens, or it could help the pathogen be acquired or escape specific tissues within the vector. For instance, *Tomato yellow leaf curl virus* (TYLCV) induces autophagy in its insect vector, the whitefly (*Bemisia tabaci*), and this induction results in decreased transmission while the inhibition of this response facilitates virus transmission [12]. In contrast, Chen, et al. [13] found that autophagy is induced by *Rice gall dwarf virus* (RGDV) in the intestine of its insect vector, the rice leafhopper (*Recilia dorsalis*). Further investigation demonstrated that the induction of autophagy improves the transmission of RGDV, whereas its inhibition blocks viral transmission. However, fewer studies have assessed the induction or inhibition of autophagy by vector-borne plant pathogenic bacteria and its potential role in bacterial transmission. This question needs to be assessed because autophagy is also induced in response to bacteria, for example, autophagy is involved regulating the population of the endosymbionts in *Drosophila* and beetles [14,15].

*Candidatus* Liberibacter solanacearum’ (Lso) is a phloem-limited, Gram-negative, unculturable bacterium [16]. It is a pathogen that infects members of the Apiaceae and Solanaceae plant families and causes great economic losses. Presently, several Lso haplotypes have been identified in the world [17,18,19,20,21,22]. Haplotypes LsoA and LsoB are transmitted by the potato psyllid (also known as tomato psyllid), *Bactericera cockerelli*, and infect numerous solanaceous plants (e.g., potatoes and tomatoes) in North America [23,24]. Lso has recently been found associated with other psyllids in the USA (e.g., psyllids associated with ash trees and *Rhamnus*) but whether they are transmitted to plants has not been established yet [25,26]. Both LsoA and LsoB are associated with potato zebra chip, a disease that has been responsible for losses of millions of dollars in potato producing regions [27]. Lso is transmitted by psyllids in a circulative and persistent manner [28,29,30]. Therefore, the psyllid gut is the first organ that Lso encounters. This organ could act as a barrier for Lso transmission and determine Lso transmission efficiency. Indeed, the ability of the bacteria to infect the gut depends on the insect immune responses as well as the bacterial strategies deployed to disrupt the host immunity.

In our previous studies, we evaluated whether Lso might induce apoptosis in psyllid guts. We did not find evidence of apoptosis in response to either LsoA or LsoB; further, we showed that Lso might repress the apoptotic response in the psyllid guts at an early stage of the infection for acquisition and transmission [31,32]. In this study, we aim to understand whether there might be an autophagic immune response in the psyllid gut following exposure to Lso. Toward this end, we first examined the potato psyllid transcriptome [33] to identify ATGs. Because there are few studies thoroughly investigating ATGs in hemipteran insects [34], we also annotated ATGs in representative Sternorrhyncha species including the pea aphid (*Acyrthosiphon pisum*), the whitefly (*B. tabaci*), and the Asian citrus psyllid (*Diaphorina citri*) which genomes have been sequenced [35,36,37,38]. Second, we evaluated the expression profiles of ATGs in response to LsoA or LsoB infection in the psyllid gut. We focused on the autophagic response of adult psyllids from infected colonies, which most likely acquired the bacteria during the nymphal stages, as well as in the responses that occur during the early infection. This study represents a first step towards understanding the molecular mechanisms and the potential role of autophagy in potato psyllid and Lso interactions.

## 2. Materials and Methods

### 2.1. Insect Colonies and Tomato Plants

Lso-free, LsoA- and LsoB-infected psyllid colonies were obtained as described in Yao, et al. [39]. These colonies were maintained separately on tomato plants (Moneymaker; Victory Seed Company, Molalla, OR, USA) in insect-proof cages (24 cm × 13.5 cm × 13.5 cm, BioQuip^®^, Compton, CA, USA) at room temperature 24 ± 2 °C and under a photoperiod of 16:8 h (L:D). 

Tomato plants infected with Lso were obtained as described in Nachappa, et al. [40]. Briefly, six-week-old tomato plants were infected using three male psyllids harboring LsoA or LsoB, respectively. One week later, the psyllids were removed from the cages and the plants were tested for Lso infection using the LsoF/OI2 primers [41] three weeks after insect removal. Further, the Lso haplotype in the plants was confirmed using the Lso SSR-1 primers [19]. 

### 2.2. Identification and Validation of Autophagy-Related Genes

Genes potentially involved in autophagy in hemipteran insects were identified through blast searches of the potato psyllid transcriptome [33] and the genome of the pea aphid, the whitefly, and the Asian citrus psyllid [35,36,37,38] using the 20 *D. melanogaster* predicted ATGs as query (e-value cut off 1 × 10^−20^) [42]. The *D. melanogaster* ATGs are from the FlyBase: https://flybase.org/reports/FBgg0000076.html (accessed on 15 January 2019). 

Specific primers for each candidate gene were designed (Table S1). RNA from a pool of 50 adult psyllids was purified using the RNeasy Mini Kit (Qiagen, Hilden, Germany), followed by a Dnase I treatment with Turbo Dnase (Ambion, Invitrogen, Waltham, MA, USA). Then, cDNA was produced using the Verso cDNA Synthesis kit (Thermo, Waltham, MA, USA) and anchored-Oligo (dT) primers according to the manufacturer’s manual. Candidate genes were amplified by PCR. The PCR conditions were 95 °C for 2 min; followed by 35 cycles of 95 °C for 30 s, 60 °C for 30 s, and 72 °C for 30–90 s (depending on the amplicon size); and a final extension at 72 °C for 5 min. Amplicons were resolved in a 1% agarose gel and they were purified using the PureLink Quick Gel Extraction kit (Invitrogen, Waltham, MA, USA). Each PCR fragment (150 ng) was cloned into the pGEM-T easy vector using the pGEM-T easy cloning kit (Promega, Madison, WI, USA) and transformed into NovaBlue Singles Competent cells (Novagen, Temecula, CA, USA). For each candidate, the plasmid from at least three colonies was sequenced by Eton Bioscience Inc. (San Diego, CA, USA). The obtained sequences were compared to the bioinformatics predictions.

### 2.3. Expression of Autophagy-Related Genes

The expression of candidate ATGs was evaluated in the psyllid gut upon Lso infection. To obtain newly infected insects, approximately one-week-old female adult psyllids from the Lso-free colony were transferred to LsoA- or LsoB-infected plants. The insects were collected 2, 3, 5, and 7 days later. Insects from the Lso-free colony were used as a control. Three replicates were analyzed for each treatment, and each replicate had 200 psyllid individuals. After exposure, the psyllid guts were dissected in 1× PBS with RNAlater (Ambion, Invitrogen, Waltham, MA, USA) under the stereomicroscope (Olympus, Tokyo, Japan) as described in Ibanez, et al. [43]. The guts of female adult psyllids from the Lso-free, LsoA- and LsoB-infected colonies were dissected as described above. RNA purification and cDNA synthesis from each sample was performed as described above. The expression of ATGs was evaluated by quantitative real-time PCR (qPCR) using the SensiFAST SYBR Hi-ROX kit (Bioline, London, UK) following the manufacturer’s manual. For each reaction, 25 ng of cDNA, 250 nM of each primer (Table S1), and 1× of SYBR Green Master Mix were mixed. The volume was adjusted to 10 μL with nuclease-free water. The qPCR conditions were as follows: 95 °C for 2 min followed by 40 cycles at 95 °C for 5 s and 60 °C for 30 s. The qPCR assays were performed using a QuantStudio™ 6 Flex Real-Time PCR System (Applied Biosystems, Foster City, CA, USA). All reactions were performed in triplicates and negative controls were included in each run. The relative expression of the candidate genes was estimated with the delta-delta CT method [44] using elongation factor-1a (GenBank KT185020) and ribosomal protein subunit 18 (GenBank KT279693) [45] as reference genes.

### 2.4. Data Analysis

All statistical analyses were conducted with JMP Version 12 (SAS Institute Inc., Cary, NC, USA). Regulation of ATGs was determined using one-way ANOVA with Tukey’s *post hoc* test.

## 3. Results

### 3.1. Autophagy-Related Genes in Potato Psyllid and Other Hemipteran Insects

A total of 19 ATG homologs were identified and their sequences were validated in the potato psyllid via transcriptome search. These genes were classified into 17 ATG gene families. The gene names and abbreviations used hereafter are listed in Table 1. In parallel, 22, 19, and 27 ATG were also identified in the genome of the pea aphid, the whitefly, and the Asian citrus psyllid, respectively (Table S2). With the exception of ATG4a, at least one putative homolog to the *Drosophila* ATGs was identified in the *B. cockerelli* transcriptome; those included serine/threonine protein kinase ULK2 (*BcATG1*), *BcATG2*-*BcATG5*, Beclin1 (*BcATG6*), *BcATG7*, gamma-aminobutyric acid receptor-associated protein (*BcATG8*), *BcATG9*, *BcATG10*, *BcATG12*, *BcATG13*, beclin 1-associated autophagy-related key regulator (*BcATG14*), *BcATG16*, RB1-inducible coiled-coil protein 1-like (*BcATG17*), WD repeat domain phosphoinositide-interacting proteins (*BcATG18*), and *BcATG101*. Further, ATGs from each *Drosophila* ATG family were identified in the pea aphid and the whitefly genomes, however, ATG12 and ATG14 were absent in the current Asian citrus psyllid genome.

### 3.2. Expression of Autophagy-Related Genes upon Lso Infection

The expression pattern of the identified potato psyllid ATGs was evaluated in the gut of adults following specific acquisition access periods on Lso-infected plants. Differences in the expression of ATGs were identified upon LsoA and LsoB acquisition.

**Figure 1:**
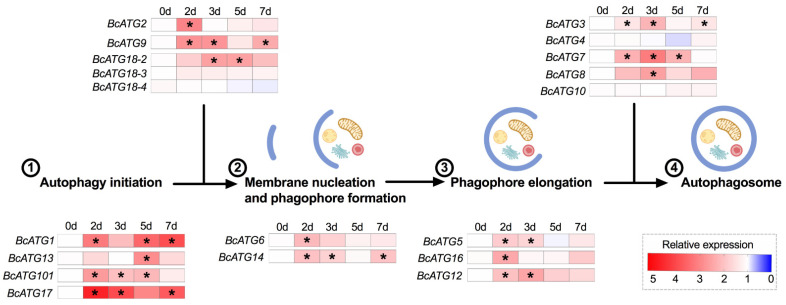
Heat map of gene expression profiles of ATGs upon LsoA infection corresponding to the process of autophagosome formation. 0 d indicates the control, in which psyllids were taken from the Lso-free colony; 2 d, 3 d, 5 d, and 7 d indicate the psyllids were infected by LsoA for 2, 3, 5, and 7 days, respectively (d indicates day). The numbers indicate the steps of autophagosome formation. The asterisks (*) indicate statistical differences compared to control at *p* < 0.05 using one-way ANOVA with Tukey’s *post hoc* test.

Upon LsoA acquisition, 15 of the 19 ATGs were regulated. Among those, *BcATG2* was only up-regulated at day 2 compared to all other time points or compared to the uninfected guts, while *BcATG6* and *BcATG16* were up-regulated at day 2 compared to the uninfected guts. *BcATG1*, *BcATG101*, *BcATG17*, *BcATG9*, *BcATG14*, *BcATG5*, *BcATG12*, *BcATG3*, and *BcATG7* remained up-regulated longer. *BcATG13*, *BcATG18-2*, *BcATG12*, *BcATG3*, *BcATG7*, and *BcATG8* were expressed at higher levels at days 3 or 5. Four genes (*BcATG18-3*, *BcATG18-4*, *BcATG4B*, and *BcATG10*) were not significantly regulated upon LsoA infection (Figure S1). Overall, a majority of genes potentially involved in the steps previous to the phagophore formation were up-regulated upon LsoA acquisition (Figure 1).

**Figure 2:**
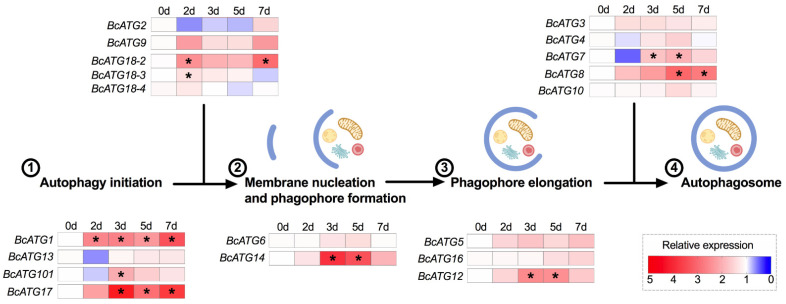
Heat map of gene expression profiles of ATGs upon LsoB infection corresponding to the process of autophagosome formation. 0 d indicates the control, in which psyllids were taken from the Lso-free colony; 2 d, 3 d, 5 d, and 7 d indicate the psyllids were infected by LsoB for 2, 3, 5, and 7 days, respectively (d indicates day). The numbers indicate the steps of autophagosome formation. The asterisks (*) indicate statistical differences compared to control at *p* < 0.05 using one-way ANOVA with Tukey’s *post hoc* test.

Upon LsoB acquisition, 10 of the 19 ATGs were regulated and multiple members had similar expression patterns. First, *BcATG18-3* was only up-regulated at day 2 compared to the uninfected guts or day 7, while *BcATG1* and *BcATG18-2* were also up-regulated at day 2 but remained up-regulated longer and/or at later time points. *BcATG101*, *BcATG17*, *BcATG14*, *BcATG12*, *BcATG7*, and *BcATG8* were expressed at higher levels at days 3 or 5. Nine genes were not significantly regulated upon LsoB infection (Figure 2 and Figure S2). 

### 3.3. Expression of Autophagy-Related Genes in Response to Persistent Lso Infection

**Figure 3:**
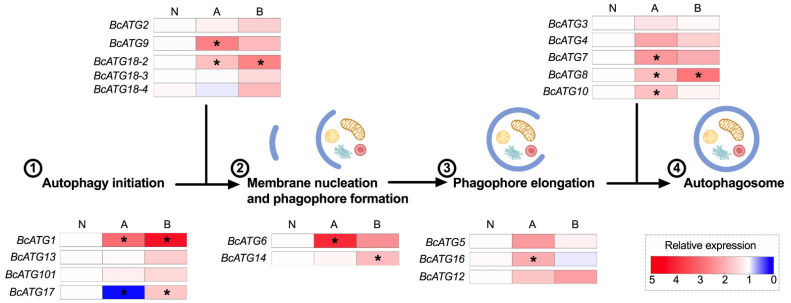
Heat map of gene expression profiles of ATGs in response to LsoA and LsoB persistent infection corresponding to the process of autophagosome formation. N: Lso-free colonies; A and B indicate the psyllids were infected by LsoA and LsoB, respectively. The numbers indicate the steps of autophagosome formation. The asterisks (*) indicate statistical differences compared to control at *p* < 0.05 using one-way ANOVA with Tukey’s *post hoc* test.

The expression pattern of the identified ATGs was evaluated in the gut of adults from the Lso-infected psyllid colonies, which most likely acquired the bacteria during the nymphal stages. We found that approximately half of the ATGs were significantly regulated in response to LsoA or LsoB infection. Eight ATG genes (*BcATG1*, *BcATG9*, *BcATG18-2*, *BcATG6*, *BcATG16*, *BcATG7*, *BcATG8*, and *BcATG10*) were significantly up-regulated in response to LsoA, while *BcATG17* was down-regulated. However, five genes (*BcATG1*, *BcATG17*, *BcATG18-2*, *BcATG14*, and *BcATG8*) were significantly up-regulated in response to LsoB. Taken together, three genes (*BcATG1*, *BcATG18-2*, and *BcATG8*) were significantly up-regulated in response to both LsoA and LsoB (Figure S3). Overall, genes across all the stages of autophagosome formation were significantly regulated (Figure 3).

## 4. Discussion

Autophagy is a self-regulated process and is highly conserved from yeast to mammals [10]. The role of autophagy is not limited to the conditions under the stimulus of starvation. It also has a significant role in innate immunity. Indeed, autophagy is a cellular degradation process that can capture and eliminate intracellular microbes by delivering them to lysosomes for destruction. In addition to directly degrading pathogens, autophagy can also activate the host immune system [46]. In mammals, autophagy provides an excellent intracellular defense system against intracellular pathogens, such as *Listeria monocytogenes* and *Shigella flexneri* [47,48]. Take *S. flexneri* for example, after bacterial uptake into host cells, the bacterial peptidoglycans can be detected by nucleotide-binding oligomerization domain (NOD)-like receptors (NLRs), which can further interact with Atg16L1 and recruit other autophagy proteins to initiate autophagosome biogenesis in response to bacterial invasion [49]. In *Drosophila*, autophagy is also an essential component of immunity. For instance, Shelly, Lukinova, Bambina, Berman and Cherry [11] found that autophagy in *Drosophila* played a direct antiviral role against the vesicular stomatitis virus (VSV). Indeed, silencing in *D. melanogaster* S2 cells of several autophagy-related genes including ATG1, ATG5, ATG8a/LC3, and ATG18/WIPI2 followed by viral challenge resulted in increased VSV infection rate. Similarly, upon VSV infection, mutant flies depleted for ATG18/WIPI2 presented increased viral replication compared to control flies as well as increased mortality when compared to unchallenged mutant flies. In addition to virus response, the studies also indicated that autophagy components are important for *D. melanogaster* defense against infection with bacteria, such as *Escherichia coli* and *Wolbachia* [14,50]. For example, RNAi knockdown of autophagy components (e.g., ATG5, ATG7, and ATG12) caused an increased pathogen load and decreased survival upon *E. coli* infection [50].

The core machinery of autophagy can be classified into several functional gene units including the ATG1/ULK1 (Uncoordinated-51 like kinase) complex (ULK1, ULK2, ATG13, ATG17, ATG101, FIP200, etc.), the transmembrane protein ATG9 cycling system (ATG2, ATG9, and ATG18), the class III phosphoinositide 3-kinase [PI(3)K] complex (ATG14L, VPS34, Beclin-1, and VPS15), and two ubiquitin-like conjugating systems, ATG12-ATG5 and ATG8/LC3 (light chain 3) [51]. The canonical autophagosome formation process can be subdivided into several phases, including autophagy initiation, membrane nucleation, phagophore formation, phagophore elongation, and autophagosome completion [52,53]. First, the ULK/ATG1 and ATG9 complex recruit the autophagy-specific PI(3)K to generate phosphatidylinositol 3-phosphate [PI(3)P] required for membrane nucleation and phagophore formation. To start elongation, the phagophore recruits PI(3)P-binding complex (ATG18/WIPI and ATG2) and then binds ATG5-ATG12/ATG16L1 complex. Finally, the accumulation of those proteins initiates the conjugation of microtubule-associated protein LC3/ATG8 family members to the phagophore and forms the autophagosome [9,53]. 

Although the ATG genes are very critical for autophagosome formation, it is now clear that ATG genes also have autophagy-independent roles in immunity [54]. A recent study in mammalian cells found that ATG8/LC3 can recruit innate immune proteins (e.g., GTPases) for a type of antiviral defense that appears to not involve fusion with the lysosome [55]. Other core machinery autophagy components, such as ULK1, FIP200, ATG14L, ATG16L1 and ATG9, are also recruited to the bacteria *Salmonella*, and each of them has a role in restricting the intracellular growth of *Salmonella* in humans [56]. These new insights would likely stimulate further analysis of autophagy and autophagy proteins in other organisms, including *Drosophila* and other insects. In particular, these studies could determine whether these proteins act in canonical autophagy, noncanonical autophagy, or some other process to provide an immune defense. 

In this study, we annotated autophagy-related genes in the potato psyllid and we evaluated their expression in the gut indeed following Lso exposure or infection. A significant change in expression was measured in response to Lso. These genes were regulated in a temporal manner showing different regulation profiles. Upon LsoA infection, most of the ATGs were significantly up-regulated at a 2-day infection, probably at a time when Lso first began to translocate into the gut cells. In particular, the ATGs involved in autophagy initiation and phagophore formation showed a more intense up-regulation profile. For instance, *BcATG1* and its subunit *BcATG17*, which control the initiation of autophagosome formation, both expressed extensive up-regulation profiles. The induction of six ATGs was delayed until day 3 or day 5 upon exposure to LsoA. Furthermore, nine genes remained induced for a longer period of time. Finally, five genes (*BcATG1*, *BcATG17*, *BcATG9*, *BcATG14*, and *BcATG3*) were still up-regulated at day 7. However, the expression of the other tested genes had been reduced to their prior levels. The transcriptional regulation of ATG genes is critical for autophagy. Indeed, the majority of the ATG genes are up-regulated upon stress [57].

The regulation of ATGs upon LsoB infection showed a different pattern compared to LsoA, and approximately half of those genes were regulated during the 7 days analyzed. Specifically, only three genes (*BcATG1*, *BcATG18-2*, and *BcATG18-3*), which contribute to the autophagy initiation and phagophore formation, were significantly up-regulated at the beginning of acquisition (2 days). The other seven genes were induced after 3 days of infection. Overall, the induction of ATGs upon LsoB infection was delayed and/or at a lower level when compared to their expression upon LsoA infection. For instance, *BcATG8*, a ubiquitin-like protein required for the formation of autophagosomal membranes, was significantly up-regulated at day 3 in response to LsoA, while it was induced after day 5 upon LsoB infection. This delayed response probably allows LsoB to be able to replicate and translocate through the gut epithelium faster. Indeed, in a previous study, we have shown that LsoB accumulates faster and to higher levels in the gut of adult psyllids. Further, this difference in accumulation correlates with reduced latency and higher transmission efficiency of LsoB than LsoA [31]. We also noted that several genes (e.g., *BcATG13*, *BcATG101*, and *BcATG2*) that function before phagophore formation showed a down-regulated profile (but not significant) upon LsoB infection, probably indicating an early repression of the autophagic response by LsoB in the psyllid gut to some extent. Indeed, although bacteria can be targeted and eliminated by autophagy, some of them have evolved strategies to escape this defense system or even hijack the autophagy machinery to promote their intracellular growth [54]. Importantly, some bacteria inhibit autophagy by directly interfering with the activity of autophagy components. For instance, *Legionella pneumophila* avoid autophagy using its effector protein by mimicking the function of ATG4B-like cysteine protease of the host that directly targets the amide bond between the tyrosine and the glycine at the C terminus of LC3/ATG8, which is covalently linked to phosphatidylethanolamine (PE) during the autophagy induction to form LC3-PE; therefore, further inhibiting autophagosome formation [58]. We hypothesize that LsoB can repress (or not induce to the same level) the autophagic response in the psyllid gut at the early stages of infection while LsoA could not. Indeed, the early LsoA infection significantly up-regulated all the ATGs involved in autophagy induction in other hemipteran insects by plant viruses. Specifically, three autophagy-related genes (*Ulk1*/*Atg1*, *Atg5*, and *Atg8*) increased rapidly and significantly after 48 h post inoculation of RGDV in the rice leafhopper [13]; furthermore, the expression of the three ATG genes (*Atg3*, *Atg9* and *Atg12*) increased significantly in TYLCV-infected whiteflies [12]. In our study, *BcATG1*, *BcATG5*, *BcATG3*, *BcATG9*, and *BcATG12* were significantly regulated after 2 days of LsoA infection, and *BcATG8* was expressed at higher levels at day 3. However, only three of those genes (*BcATG1*, *BcATG12*, and *BcATG8*) were induced upon LsoB infection. Therefore, we hypothesized that autophagy could be induced in the gut of the adult psyllid upon LsoA infection, and this induction may be linked to the reduced increase of LsoA titer in the gut of psyllids compared to LsoB and its delayed transmission. It is unclear whether autophagy is induced in response to LsoB. If induced, this response is probably delayed compared to the induction by LsoA. 

In addition, from the ATGs expression profile in response to persistent Lso infection, we also found that several ATGs were up-regulated in response to LsoA or LsoB and that more ATGs were up-regulated in response to LsoA. This observation is consistent with the ATGs induction upon Lso infection. Importantly, differences in virulence between these two haplotypes were determined in association with their host plants and insect vector: in both cases, LsoB was found to be more pathogenic [39,59,60]. It is, therefore, possible that LsoB is able to better defend itself against the psyllid immunity, or even also manipulate those defenses to its advantage. We also noted that *BcATG17* was significantly down-regulated in response to persistent LsoA infection, indicating a putative repression of the autophagic response. However, both LsoA and LsoB significantly induced the core autophagy protein, *BcATG8*. Therefore, we hypothesized that autophagy could be occurring in the gut of the adult psyllid in response to persistent LsoA and LsoB infection. However, the autophagic immune response cannot be an efficient defense because both LsoA and LsoB can be successfully transmitted by potato psyllids. The plausible explanation is that even if autophagy is induced in response to the pathogen, this defense strategy is not sufficient or not widespread enough to stop Lso.

## 5. Conclusions

In summary, we identified 19 homologs of autophagy-related genes from potato psyllid. Our study suggested that the expression of the majority of autophagy-related genes changed significantly after LsoA or LsoB exposure or in the case of established infection of the gut. Importantly, in the case of autophagy, the gene expression correlates with the actual autophagy [10]. Further investigations need to be conducted to deepen the study of the autophagic response of potato psyllid to Lso infection and its role in Lso transmission. A combination of methods, such as western blotting, immunostaining and transmission electron microscopy, as well as RNAi-mediated gene silencing to analyze the autophagic response are recommended.

## Figures and Tables

**Table 1:** Name of *B. cockerelli* autophagy-related genes and their codes used in this paper.

Gene Name	Code
Serine/threonine-protein kinase ULK2-like isoform 1	*BcATG1*
Autophagy-related protein 2-like	*BcATG2*
Autophagy related protein Atg3-like protein	*BcATG3*
Cysteine protease ATG4B-like isoform 1	*BcATG4B*
Autophagy protein 5	*BcATG5*
Beclin-1-like protein	*BcATG6*
Ubiquitin-like modifier-activating enzyme ATG7	*BcATG7*
Gamma-aminobutyric acid receptor-associated protein	*BcATG8*
Autophagy-related protein 9A	*BcATG9*
Ubiquitin-like-conjugating enzyme ATG10	*BcATG10*
Autophagy protein 12-like	*BcATG12*
Autophagy-related protein 13 homolog	*BcATG13*
Beclin 1-associated autophagy-related key regulator-like	*BcATG14*
Autophagy-related protein 16-1-like	*BcATG16*
RB1-inducible coiled-coil protein 1-like	*BcATG17*
WD repeat domain phosphoinositide-interacting protein 2	*BcATG18-2*
WD repeat domain phosphoinositide-interacting protein 3	*BcATG18-3*
WD repeat domain phosphoinositide-interacting protein 4	*BcATG18-4*
Autophagy-related protein 101-like isoform 1	*BcATG101*

## Data Availability

Not applicable.

## Supplementary Materials

The following are available online at https://www.mdpi.com/article/10.3390/insects12121073/s1, Figure S1: Regulation of ATGs in the psyllid gut upon LsoA infection. Data represent means ± SD of three independent experiments. Different letters indicate statistical differences at *p* < 0.05 using one-way ANOVA with Tukey’s *post hoc* test. N: Lso-free colonies; 2 d, 3 d, 5 d, and 7 d indicate the psyllids were infected by LsoA for 2, 3, 5, and 7 days, respectively. The numbers indicate the steps of autophagosome formation. Figure S2: Regulation of ATGs in the psyllid gut upon LsoB infection. Data represent means ± SD of three independent experiments. Different letters indicate statistical differences at *p* < 0.05 using one-way ANOVA with Tukey’s *post hoc* test. N: Lso-free colonies; 2 d, 3 d, 5 d, and 7 d indicate the psyllids were infected by LsoB for 2, 3, 5, and 7 days, respectively. The numbers indicate the steps of autophagosome formation. Figure S3: Regulation of ATGs in the psyllid gut in response to LsoA and LsoB persistent infection. Data represent means ± SD of three independent experiments. Different letters indicate statistical differences at *p* < 0.05 using one-way ANOVA with Tukey’s *post hoc* test. N: Lso-free colonies; A and B indicate the psyllids were infected by LsoA and LsoB, respectively. The numbers indicate the steps of autophagosome formation. Table S1: Primers for bioinformatics validation and gene expression analyses by qPCR of autophagy-related genes. Table S2: Comparison of autophagy-related genes in *D. melanogaster*, *A. pisum*, *B. tabaci*, *D. citri* and *B. cockerelli*.
