# Supplementary material for: Identification of Autophagy-Related Genes in the Potato Psyllid, Bactericera cockerelli and Their Expression Profile in Response to ‘Candidatus Liberibacter Solanacearum’ in the Gut
Source: Insects. 2021 Nov 30;12(12):1073. doi: 10.3390/insects12121073 (PMC8708441; doi:10.3390/insects12121073)
Supplement: Supplementary file 1 [file insects-12-01073-s001.zip › insects-1450756-supplementary.pdf]

Table S1. Primers for bioinformatics validation and gene expression analyses by qPCR of autophagy-related genes.

Gene Code	Validation Primers (5'-3')	qPCR primers (5'-3')
BcATG1	F: AACTCTCCCCTCACCCTCAT	F: CGACCCACAACAACAACAAC
	R: GTCTACATGGCGGAGGTGTT	R: ATTTAGCCAGCGTTTCACAG
BcATG2	F: TAAAACACCACCGGAGGAAG	F: CAAGGACAGTGAAGGAGCAG
	R: GCTCACGCTAGGAATCGAAC	R: TACCTGGACACGGAGAACAC
BcATG3	F: AGTTTGTGGCAGCAGGAGAT	F: CTCAAGACGCTGCAAACAAA
	R: AAAGCGGTTTAGAACCGAAA	R: GTGTCAACCCAACCCTCATC
BcATG4	F: TTAGCCCTTTGGTATTGTGA	F: TCCAAGACATCAACCCTGTG
	R: GCGAAGAGGCTGTAAGTATC	R: GCTTTCCCCCTATCACTCCT
BcATG5	F: CACCGAGAGATCACACGAAA	F: ATGTGACCGACCATCAGCTT
	R: GGTGTTGGAGTTGCCTTCAT	R: TCCACAGCTTCTCTGCTAGGAC
BcATG6	F: CACTACCCCACACTCAATAACA	F: ATTCACCATCTCCTCTCCA
	R: CATCTGGAAGAGGAAAGCAT	R: TGACTCTCTGCTTCTACACTGA
BcATG7	F: CGGTTCTTTCACCAGCTCTC	F: ATGACGGGGACACAAAGTTC
	R: GAGACTGACGAGCAGGGTTC	R: GATCCATAGACTCCCGCAAG
BcATG8	F: ATCAACGTTAAGAGCTCAAG	F: GTTGAAAAAGCCCCCAAAG
	R: CAGGGTCCAGGGTAGAAAGA	R: GCGGATGAGGAAATAGAACTG
BcATG9	F: TCCTCAGGAAGGAGGAGTGA	F: GACAAGCACACACATCCAGAG
	R: AGGAGGACTTGGGAGCTAGG	R: GAGTTACAAGTGGCGAGCATAA
BcATG10	F: CTACATGAAACCCCATAAGA	F: CACCCCTATCTGAGGAAACC
	R: TCTCTGCCTCTTAACCCATA	R: CTTGACCCTTGTTGGTAACG
BcATG12	F: GTACCGATTGACTTGCATTC	F: TTTGCACCTTCTCCTGACAC
	R: ATCCACTTCCTCCATGATTG	R: CCATGCTTGTGTTTTGCAGT
BcATG13	F: TATTGTGCGTCCCATGTGTT	F: GGATGGCTTCAACAAGGTTC
	R: GATGGTCAGGTCCGTTCTGT	R: GATGGTCAGGTCCGTTCTGT
BcATG14	F: AACAGCTTCGAGCTAAGCAA	F: TAAATTCAGCAAGCGTGTGG
	R: GCTCTCCTCCTCCTCATCCT	R: ATGCAAAGGCAGGTCAGTGT

BcATG16	F: TGGATGTGTAGAGGCAGGAA<br>R: AGCAAGACTGCTGGAGAAGG	F: GCTGTGATAGAACCCCCAAG<br>R: CGTCGTATGTCACCAGATCG
BcATG17	F:<br>R:	F: CAGAAGGCACTGATACGGAAG<br>R: AATGTTCGTCCTCACGGTCT
BcATG18-2	F: TTTCAAGTGCTGCTGTAGAT<br>R: AGGCGAGATGTTTGTTGCTT	F: GCATAGGTTGGATGGTCGTAA<br>R: TCGGAAGTTTTGAGAGAGCA
BcATG18-3	F: CACAAGTCTGAAAGTGGAAAGC<br>R: GGGAATGCGAGGAGAGAGT	F: CTAGGCCATGTGGAAATGCT<br>R: TGTTGGGGTACTTTGGGTGT
BcATG18-4	F: ACCTTTGCCTTCTAAATGAG<br>R: TGTCTCTTCTCAATCTTACAGC	F: CAAGCAACATGAGCAAAGGA<br>R: GCCATTATCCATGCAACAAG
BcATG101	F: GGTCACCAAATCGACGAAGT<br>R: TAGTTGATGGGGTCGTAGGC	F: CTCAAGCCTCTTTCACACGA<br>R: AGGGTGCCAACGGAGTATTT

Table S2. Comparison of autophagy-related genes in *D. melanogaster, A. pisum, B. tabaci, D. citri*, and *B. cockerelli.*

***D. melanogaster* (conserved domains, protein length in AA)**	***A. pisum* (protein length in AA)**	***B. tabaci* (protein length in AA)**	***D. citri* (protein length in AA)**	***B. cockerelli* (most similar protein, E-value, protein length in AA)**
Autophagy-related 1 (Atg1) (855 AA)	LOC100159542 serine/threonine-protein kinase unc-51 (663 AA)	LOC109038743 serine/threonine-protein kinase ULK2 (664 AA)	LOC103514260 serine/threonine -protein kinase ULK1 (567 AA)	serine/threonine-protein kinase ULK2-like isoform 1 (2.72112E-39, 226 AA)
Autophagy-related 2 (Atg2) (1906 AA)	LOC100169298 autophagy-related protein 2 homolog B (1983 AA)	LOC109044529 autophagy-related protein 2 homolog B isoform X1 (1993 AA) / LOC109044529 autophagy-related protein 2 homolog B isoform X2 (1964 AA)	LOC103519534 autophagy-related protein 2 homolog B (340 AA)	autophagy-related protein 2 homolog A (1.2937E-110, 1025 AA)

			LOC113465363 autophagy-related protein 2 homolog A-like (361 AA)	
			LOC103509283 autophagy-related protein 2 homolog A-like (137 AA)	
			LOC113471576 autophagy-related protein 2 homolog B-like (95 AA)	
Autophagy-related 3 (Atg3) (330 AA)	LOC100168321 autophagy related protein Aut1-like (339 AA)	LOC109031323 ubiquitin-like-conjugating enzyme ATG3 (316 AA)	LOC103517262 ubiquitin-like-conjugating enzyme ATG3 (140 AA)	autophagy related protein Atg3-like protein (4.03526E-132, 326 AA)
	LOC100159720 ubiquitin-like-conjugating enzyme ATG3 (335 AA)		LOC113470558 ubiquitin-like-conjugating enzyme ATG3 (119 AA)	
			LOC103517272 ubiquitin-like-conjugating enzyme ATG3 (89 AA)	
Autophagy-related 4a (Atg4a) (410 AA)		LOC109042782 cysteine protease ATG4A (396 AA)	LOC103520402 cysteine protease ATG4A-like (170 AA)	
			LOC103520210 cysteine protease	

			ATG4A-like (117 AA)	
Autophagy-related 4b (Atg4b) (668 AA)	LOC100160112 cysteine protease ATG4B (402 AA)		LOC103520393 cysteine protease ATG4B-like (124 AA)	cysteine protease ATG4B-like isoform 1 (3.00685E-158, 395 AA)
	LOC100571688 cysteine protease ATG4B (252 AA)			
Autophagy-related 5 (Atg5) (269 AA)	Autophagy-specific gene 5 (266 AA)	LOC109038543 autophagy protein 5 isoform X1 (289 AA)/ LOC109038543 autophagy protein 5 isoform X2 (252 AA)	LOC103512211 autophagy protein 5 (270 AA)	Autophagy protein 5 (1.11789E-124, 267 A)
Autophagy-related 6 (Atg6) (422 AA)	LOC100162879 beclin-1-like protein (420 AA)	LOC109036018 beclin-1-like protein (429 AA)	LOC103511670 beclin-1-like protein (497 AA)	Beclin-1-like protein (1.26527E-75, 519 AA)
Autophagy-related 7 (Atg7) (510 AA)	LOC100168214 ubiquitin-like modifier-activating enzyme ATG7 (680 AA)	LOC109037754 ubiquitin-like modifier-activating enzyme ATG7 (654 AA)	LOC103514245 ubiquitin-like modifier-activating enzyme ATG7 (626 AA)	Ubiquitin-like modifier-activating enzyme ATG7 (1.55145E-145, 536 AA)
Autophagy-related 8a (Atg8a) (121 AA)	LOC100163628 gamma-aminobutyric acid receptor-associated protein-like (118 AA)	LOC109035081 gamma-aminobutyric acid receptor-associated protein (120 AA)	LOC103508318 gamma-aminobutyric acid receptor-associated protein isoform X3 (120 AA)	gamma-aminobutyric acid receptor-associated protein (3E-114, 119 AA)
Autophagy-related 8b (Atg8b) (120 AA)			LOC103521693 gamma-aminobutyric acid receptor-associated protein-like (109 AA)	

Autophagy-related 9 (Atg9) (845 AA)	LOC100159271 autophagy-related protein 9A (712 AA)	LOC109034026 autophagy-related protein 9A (730 AA)	LOC103522855 autophagy-related protein 9A (606 AA)	Autophagy-related protein 9A (6.45637E-174, 717 AA)
	LOC100167077 autophagy-related protein 9A (615 AA)			
Autophagy-related 10 (Atg10) (172 AA)	LOC100575896 ubiquitin-like-conjugating enzyme ATG10 (165 AA)	LOC109040219 ubiquitin-like-conjugating enzyme ATG10 (203 AA)	LOC103523617 ubiquitin-like-conjugating enzyme ATG10 (206 AA)	ubiquitin-like-conjugating enzyme ATG10 (4E-53, 222 AA)
Autophagy-related 12 (Atg12) (111 AA)	LOC100159402 ubiquitin-like protein ATG12 (107 AA)	LOC109033666 autophagy protein 12-like (118 AA)		autophagy protein 12-like (5E-38, 113 AA)
Autophagy-related 13 (Atg13) (523 AA)	LOC100573619 autophagy-related protein 13 homolog isoform X3 (472 AA) / LOC100573619 autophagy-related protein 13 homolog isoform X1 (517 AA) / LOC100573619 autophagy-related protein 13 homolog isoform X2 (514 AA)	LOC109038021 autophagy-related protein 13 homolog isoform X1 (420 AA) / LOC109038021 autophagy-related protein 13 homolog isoform X2 (417 AA)	LOC103506104 autophagy-related protein 13-like (219 AA)	autophagy-related protein 13 homolog (3.03445E-79, 298 AA)
			LOC103515171 autophagy-related protein 13 homolog (292 AA)	
Autophagy-related 14 (Atg14) (503 AA)	LOC100164156 beclin 1-associated autophagy-related	LOC109042483 beclin 1-associated autophagy-related key regulator isoform X1 (477 AA) /		beclin 1-associated autophagy-related key regulator-like (1.86016E-81, 416AA)

	key regulator isoform X2 (483 AA) / LOC100164156 beclin 1-associated autophagy-related key regulator isoform X1 (487 AA)	LOC109042483 beclin 1-associated autophagy-related key regulator isoform X2 (476 AA)		
Autophagy-related 16 (Atg16) (612 AA)	LOC100167285 autophagy-related protein 16-1 (541 AA)	LOC109034579 autophagy-related protein 16-1 (549 AA)	LOC103513712 autophagy-related protein 16-1-like isoform X2 (344 AA) / LOC103513712 autophagy-related protein 16-1-like isoform X1 (425 AA)	Autophagy-related protein 16-1-like (0.0, 550 AA)
Autophagy-related 17 (Atg17) (1357 AA)	LOC100573296 RB1-inducible coiled-coil protein 1 isoform X1 (1039 AA) / LOC100573296 RB1-inducible coiled-coil protein 1 isoform X2 (1037 AA)	LOC109034605 RB1-inducible coiled-coil protein 1 isoform X2 (1113 AA) / LOC109034605 RB1-inducible coiled-coil protein 1 isoform X1 (1114 AA)	LOC103507010 RB1-inducible coiled-coil protein 1-like (441 AA)	RB1-inducible coiled-coil protein 1-like (0.0, 1033 AA) *
			LOC103520418 RB1-inducible coiled-coil protein 1-like (462 AA)	
Autophagy-related 18a (Atg18a) (377 AA)	LOC100164804 WD repeat domain phosphoinositide-interacting protein 3 (346 AA)	LOC109031531 WD repeat domain phosphoinositide-interacting protein 3 (348 AA)	LOC103512994 WD repeat domain phosphoinositide-interacting	WD repeat domain phosphoinositide-interacting protein 3 (6.74028E-87, 177 AA)

			protein 2-like (285 AA)	
		LOC109032757 WD repeat domain phosphoinositide-interacting protein 4-like (350 AA)	LOC103521300 WD repeat domain phosphoinositide-interacting protein 3 (349 AA)	WD repeat domain phosphoinositide-interacting protein 4-like (8.45801E-39, 106 AA)
Autophagy-related 18b (Atg18b) (471 AA)	LOC100165258 WD repeat domain phosphoinositide-interacting protein 2 isoform X3 (463 AA) / LOC100165258 WD repeat domain phosphoinositide-interacting protein 2 isoform X5 (422 AA) / LOC100165258 WD repeat domain phosphoinositide-interacting protein 2 isoform X4 (445 AA) / LOC100165258 WD repeat domain phosphoinositide-interacting protein 2 isoform X2 (464 AA)	LOC109043586 WD repeat domain phosphoinositide-interacting protein 2 isoform X2 (425 AA) / LOC109043586 WD repeat domain phosphoinositide-interacting protein 2 isoform X1 (446 AA)		WD repeat domain phosphoinositide-interacting protein 2-like (0, 465 AA)
Autophagy-related 101 (Atg101) (218 AA)	LOC100164875 autophagy-related protein 101 isoform X3 (219 AA)	LOC109034734 autophagy-related protein 101 isoform X2 (218 AA) / LOC109034734 autophagy-related protein 101 isoform X1 (232 AA)	LOC103509748 autophagy-related protein 101 (244 AA)	autophagy-related protein 101-like isoform 1 (5.64157E-82, 244 AA)

			LOC103509752 autophagy-related protein 101 (211 AA)	

* Not verified by sequencing

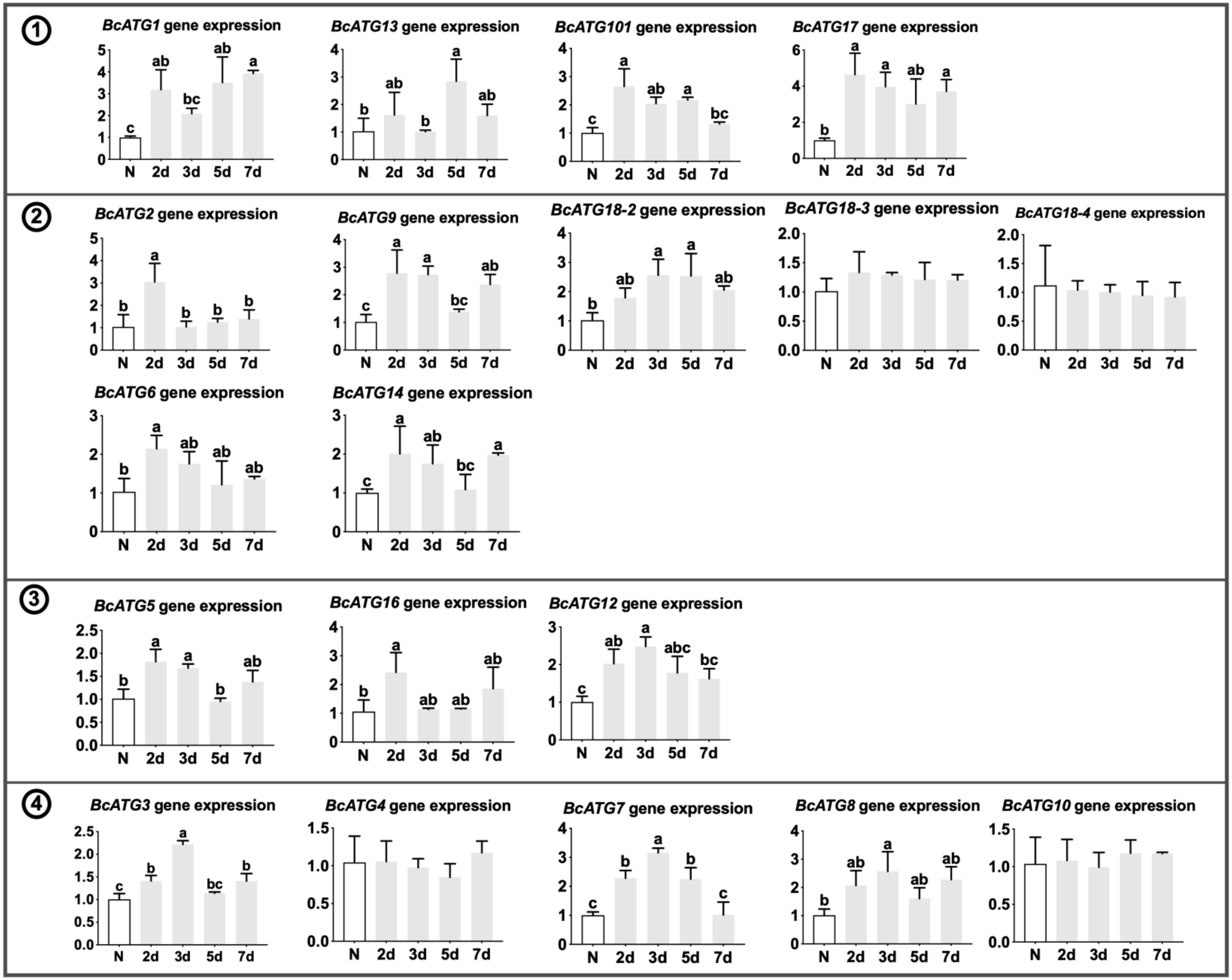

1
BcATG1 gene expression
BcATG13 gene expression
BcATG101 gene expression
BcATG17 gene expression
2
BcATG2 gene expression
BcATG9 gene expression
BcATG18-2 gene expression
BcATG18-3 gene expression
BcATG18-4 gene expression
BcATG6 gene expression
BcATG14 gene expression
3
BcATG5 gene expression
BcATG16 gene expression
BcATG12 gene expression
4
BcATG3 gene expression
BcATG4 gene expression
BcATG7 gene expression
BcATG8 gene expression
BcATG10 gene expression
N 2d 3d 5d 7d

Figure S1. Regulation of ATGs in the psyllid gut upon LsoA infection. Data represent means ± SD of three independent experiments. Different letters indicate statistical differences at $P < 0.05$ using one-way ANOVA with Tukey's *post hoc* test. N: Lso-free colonies; A-2d, A-3d, A-5d, and A-7d indicate the psyllids were infected by LsoA for 2, 3, 5, and 7 days, respectively.

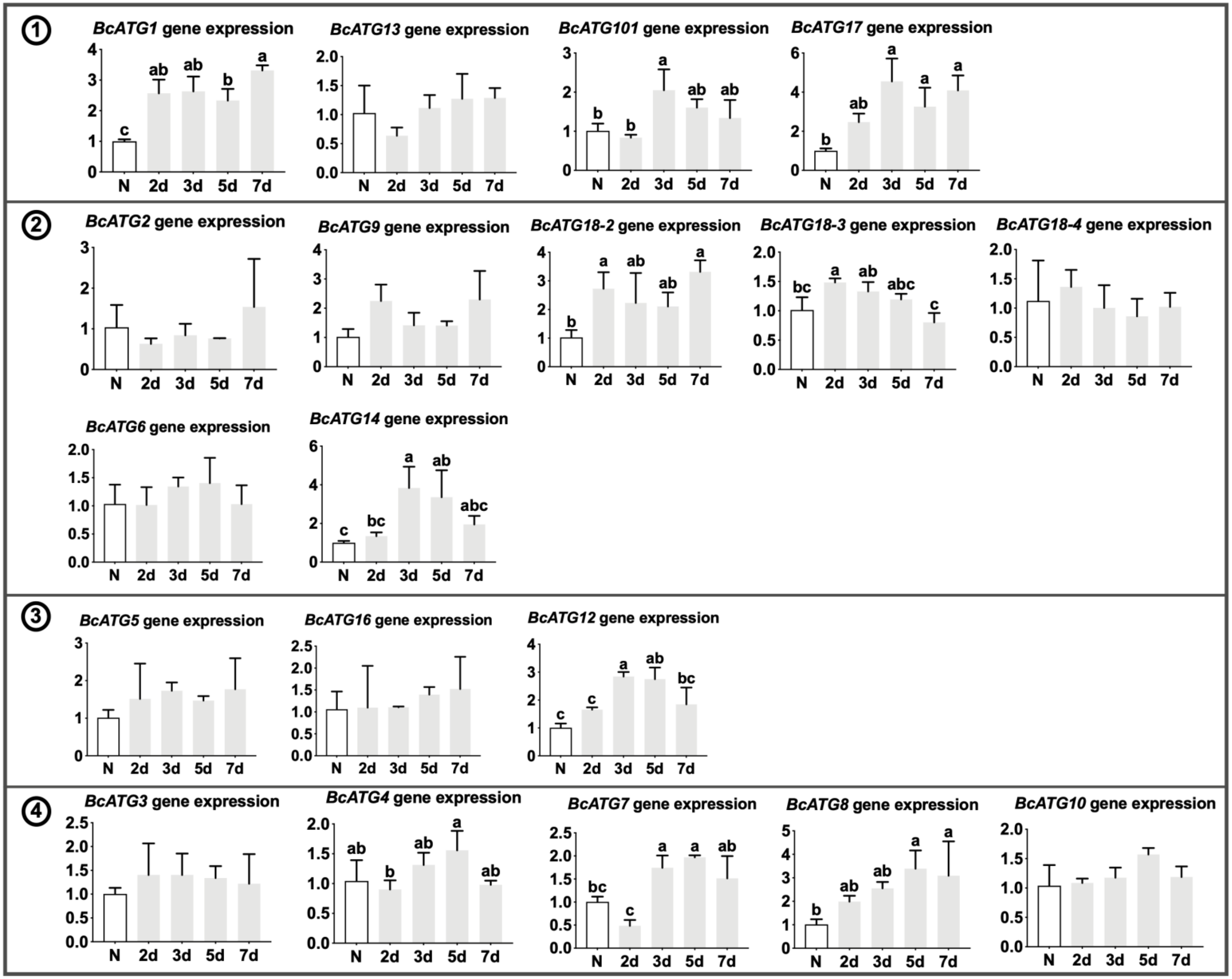
①
BcATG1 gene expression
BcATG13 gene expression
BcATG101 gene expression
BcATG17 gene expression
②
BcATG2 gene expression
BcATG9 gene expression
BcATG18-2 gene expression
BcATG18-3 gene expression
BcATG18-4 gene expression
BcATG6 gene expression
BcATG14 gene expression
③
BcATG5 gene expression
BcATG16 gene expression
BcATG12 gene expression
④
BcATG3 gene expression
BcATG4 gene expression
BcATG7 gene expression
BcATG8 gene expression
BcATG10 gene expression
N
2d
3d
5d
7d

Figure S2. Regulation of ATGs in the psyllid gut upon LsoB infection. Data represent means ± SD of three independent experiments. Different letters indicate statistical differences at $P < 0.05$ using one-way ANOVA with Tukey's *post hoc* test. N: Lso-free colonies; B-2d, B-3d, B-5d, and B-7d indicate the psyllids were infected by LsoB for 2, 3, 5, and 7 days, respectively.

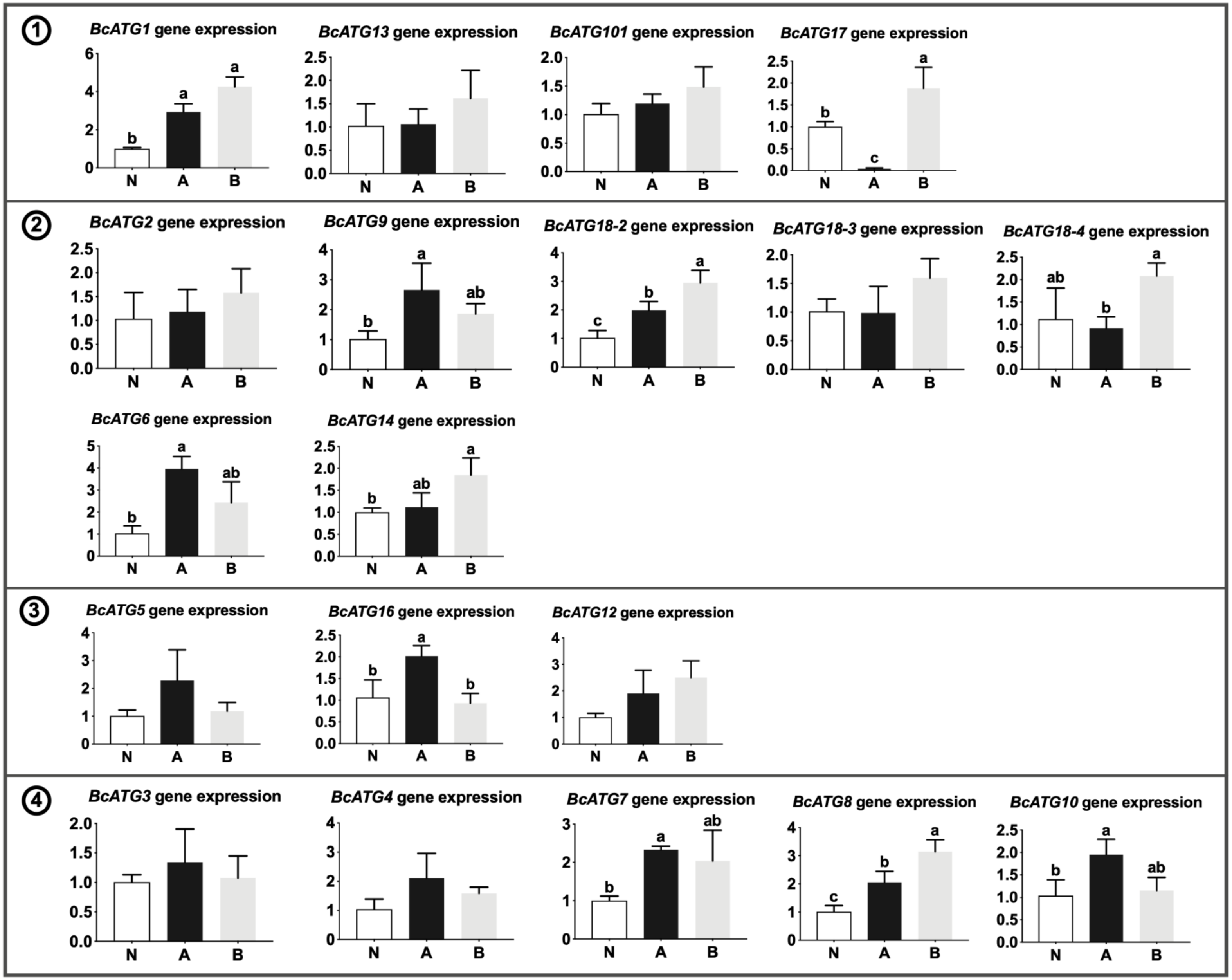
①
BcATG1 gene expression
BcATG13 gene expression
BcATG101 gene expression
BcATG17 gene expression
②
BcATG2 gene expression
BcATG9 gene expression
BcATG18-2 gene expression
BcATG18-3 gene expression
BcATG18-4 gene expression
BcATG6 gene expression
BcATG14 gene expression
③
BcATG5 gene expression
BcATG16 gene expression
BcATG12 gene expression
④
BcATG3 gene expression
BcATG4 gene expression
BcATG7 gene expression
BcATG8 gene expression
BcATG10 gene expression
N
A
B

Figure S3. Regulation of ATGs in the psyllid gut in response to LsoA and LsoB persistent infection. Data represent means ± SD of three independent experiments. Different letters indicate statistical differences at $P < 0.05$ using one-way ANOVA with Tukey's *post hoc* test. N: Lso-free colonies; A and B indicate the psyllids were infected by LsoA and LsoB, respectively.
